# Prediction of Recurrent Emergency Department Visits among Patients with Crohn’s Disease: A Retrospective Study

**DOI:** 10.3390/jcm9113651

**Published:** 2020-11-13

**Authors:** Hussein Mahajna, Yiftach Barash, Bella Ungar, Shelly Soffer, Ahmad Albshesh, Asaf Levartovsky, Shomron Ben-Horin, Eyal Klang, Uri Kopylov

**Affiliations:** 1 Department of Gastroenterology, Sheba Medical Center, Tel-Hashomer, Israel Affiliated to Sackler Faculty of Medicine, Tel-Aviv University, Tel Aviv-Yafo 52621, Israel; bella.geyshis.ungar@gmail.com (B.U.); ahmad.albshesh@gmail.com (A.A.); alavra@gmail.com (A.L.); shomron.benhorin@gmail.com (S.B.-H.); ukopylov@gmail.com (U.K.); 2 Department of Diagnostic Imaging, Sheba Medical Center, Tel Hashomer, Israel, Affiliated to Tel-Aviv University, Tel Aviv-Yafo 52621, Israel; yibarash@gmail.com (Y.B.); soffer.shelly@gmail.com (S.S.); eyalkla@hotmail.com (E.K.); 3 Deep Vision Lab, Sheba Medical Center, Affiliated to Tel-Aviv University, Tel Aviv-Yafo 52621, Israel

**Keywords:** Crohn’s disease, emergency department, recurrent visits

## Abstract

Patients with Crohn’s disease (CD) are frequently subject to symptoms causing them to seek medical care in emergency departments (ED). Recurrent ED visits are frequent after initial discharge. We aimed to identify the characteristics of patients with Crohn’s who tend to have recurrent visits to the ED. We created an electronic data repository of all patients with inflammatory bowel diseases who visited the ED in our tertiary medical center during the period 2012–2018. For this study, we retrieved consecutive Crohn’s patients who presented with CD-related symptoms to the ED and were eventually discharged. Patients who returned to the ED in 7 and 30 days were compared with those who did not. Overall, 2299 patients visited our ED with complaints related to Crohn’s disease exacerbation or complication. A total of 1259 (60% of the adult patients) were admitted for hospitalization. Of the 632 (33%) who were discharged from the ED, 53 (8.4%) and 110 (17.4%) re-visited the ED, in 7 and 30 days from discharge, respectively. In multivariable analysis, tachycardia (odds ratio (OR) = 2.19, 95% confidence interval (CI): 1.11–4.33, *p* value = 0.02), elevated alkaline phosphatase (OR = 2.09, 95% CI: 1.07–4.07, *p* value = 0.02), and hyponatremia (OR = 2.52, 95% CI: 1.24–5.10, *p* value = 0.01) were associated with revisiting the ED within 7 days. Tachycardia (OR 2.88 (95% CI 1.33–6.2)), anemia (OR 2.44 (95% CI 1.24–4.8)), and elevated alkaline phosphatase (OR 2.68 (95% CI 1.25–5.78)) were independently associated with ED returns in 30 days. Knowing these risk factors may assist in minimizing the burden of recurrent ED visits among patients with CD.

## 1. Introduction

Crohn’s disease (CD) is a chronic disease characterized by transmural inflammation throughout the gastrointestinal tract. It is characterized by a chronic course with relapses and remissions, with intestinal and extra intestinal manifestations. CD carries the potential of several intestinal complications including strictures, fistulas and abscesses. The exact etiology of CD remains unknown, and is thought to be multifactorial involving genetic, environmental factors [1]. The incidence of CD has been rising in the last few decades, especially in newly industrialized countries [2,3,4]. A similar increase in the burden of this disease was noted on health systems [5,6].

The introduction of biologic treatments in the last three decades has dramatically changed the natural history of the disease [7,8,9]. Nevertheless, many patients with CD experience exacerbations and complications of the disease. According to the literature, at least 11.3% of patients with inflammatory bowel diseases (IBD) seek acute care in the emergency department every year, with at least half of them hospitalized [10,11].

Several studies have previously investigated the burden and rates of emergency department (ED) visits by patients with IBD and found some inconsistency regarding trends in ED use among IBD patients. While some reported high and stable trends of ED utilization [11,12], a study conducted in South Korea reported that the proportion of patients with CD visiting the ED declined from 19.2% in 2007 to 11.3% in 2014, notably with the incline in the use of biologics from 0.0% to 19.2% [10].

Many patients who are being discharged home after ED visits re-visit the ED thereafter because of unresolved complaints. The recurrent attendance of the ED carries a detrimental impact in terms of economic cost, psychological stress and physical burden, as well as a delay in the diagnosis of exacerbations and complications. There are hitherto no studies aiming to characterize patients with CD who are at risk of returning to ED after being discharged. We aimed at defining the risk factors associated with recurrent visits to the ED.

## 2. Experimental Section

We created an electronic data repository of all patients with CD who visited the ED in Chaim Sheba Medical Center (a tertiary academic medical center in Ramat Gan, Israel) in the time frame between 2012 and 2018. For this study, we retrieved consecutive patients with CD who were discharged from the ED.

We included patients with CD aged above 18 years old, who presented with complaints attributed to CD exacerbation or complications and discharged home after investigation. We included the complaints: abdominal pain, diarrhea, nausea, vomiting, hematochezia, weakness and fever. We excluded complaints that are not related to the CD such as accidents, cardiac and respiratory symptoms, and infections that are not related to the gastrointestinal tract.

We excluded pregnant patients as this group of patients tend to visit the ED because of obstetric reasons and might influence the results in a manner that will limits their generalizability. We also excluded patients younger than 18 years old because of the different differential diagnosis and management and hospitalization practices between the adult and pediatric population. To avoid overexpression of frequently recurrent visits of the same patient for the same complaint in the data, we included only a single recurrent visit within 90 days and removed any additional visits that occurred within this timeframe. The collected data included demographic, clinical and laboratory data, as well as data regarding the medical treatment which is known to alter the clinical course of Crohn’s disease and was of high importance to examine. Furthermore, we sought to find out whether the timing of ED visit is associated with the risk of re-visiting the ED. Patients who re-visited the ED within 7 and 30 days were compared with those who did not. These time periods have been used in similar literature from other fields. Moreover, we hypothesized that while re-visits within 7 days represent an unresolved acute event, re-visits within 30 days includes more cases of chronic uncontrolled disease.

### 2.1. Statistical Analysis

Categorical variables were summarized as frequency and percentage. Continuous variables were evaluated for normal distribution using histogram and quantile-quantile plots. Normally distributed continuous variables were reported as means and standard deviations, while non-normally distributed continuous variables were described as medians and interquartile ranges.

The association between re-visiting the ED and categorical variables was studied using the Chi-square or Fisher exact test as appropriately needed. The association between continuous variables and re-visiting the ED was assessed using the independent samples T-test or Mann–Whitney test as appropriate.

Multiple imputation was used to treat missing values. Missing values were imputed five times and the analysis was pooled and reported as a summarized result. Linear regression was used to impute continuous variables and logistic regression was used to impute categorical variables.

Logistic regression was used for multivariable analysis. Variables which were found to be significantly associated with recurrent ED visits at the univariate analysis were included in the multivariable analysis. A backward method using the Wald test as a criterion (*p* > 0.1 for removal) was applied for variable selection.

All statistical tests were 2-sided and *p* < 0.05 was considered as statistically significant.

Statistical analysis was done using IBM SPSS Statistics for Windows, Version 23.0, Armonk, NY: IBM Corp.

### 2.2. Ethical Approval

The study was approved by the institutional review board of the Sheba Medical Center. Informed consent was waived due to the retrospective nature of the study.

## 3. Results

**Figure 1:**
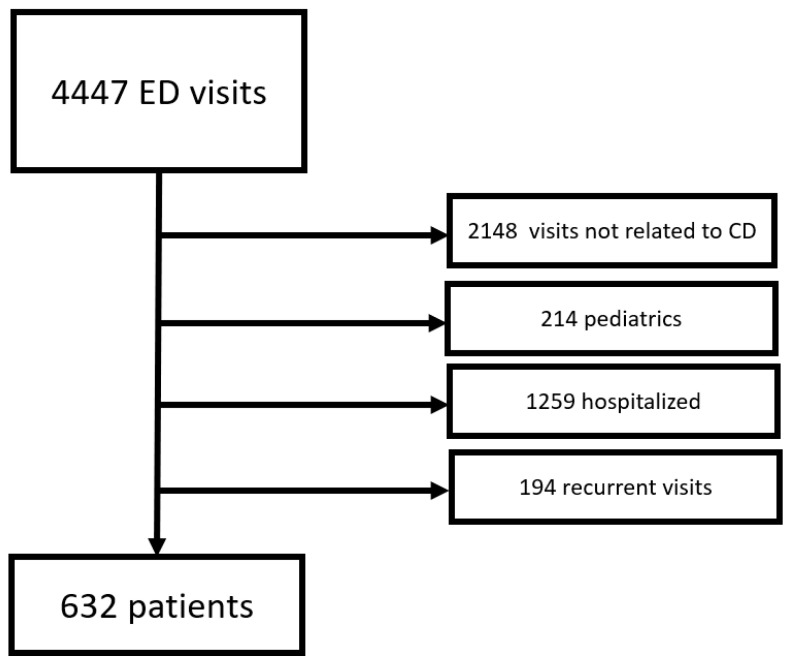
Flow chart showing the screened, included and excluded patients.

A total of 4447 patients with CD visited the ED during the study period. We excluded 2148 patients’ visits because the complaint or the diagnosis was not related to Crohn’s disease. We excluded 214 patients for being under 18 years old, 1259 patients for being hospitalized, 194 cases due to frequent visits to the ED in the preceding 90 days and 63 cases due to insufficient data. The remaining study population was 632 patients with CD who visited the ED during the study period with potentially CD-related complaints and were discharged home after workup (Figure 1).

The median age of the study population was 36 (interquartile ranges 26.0, 48.7). Two hundred seventy-nine patients (44.1%) were males. One hundred twenty-two patients (19.3%) were treated with biological treatment. Table 1 presents the characteristics of the study population.

### 3.1. Re-Visiting ED within 7 Days

During the consecutive seven days after discharge, 53 (8.4%) of patients revisited the ED. Male gender (*p* value = 0.04), elevated alkaline phosphatase (>120 IU/L) (*p* value = 0.01), hyponatremia (serum sodium < 135 meq/L) (*p* value < 0.01), and tachycardia (heart rate > 100 bpm) (*p* value < 0.01) were associated with revisiting the ED within 7 days from discharge (Table 1).

On multivariable analysis, hyponatremia (odds ratio (OR) = 2.52, 95% confidence interval (CI): 1.24–5.10, *p* value = 0.01), tachycardia (OR = 2.19, 95% CI: 1.11–4.33, *p* value = 0.02) and elevated alkaline phosphatase (OR = 2.09, 95% CI: 1.07–4.07, *p* value = 0.02) were associated with revisiting the ED within 7 days (Table 2).

### 3.2. Re-Visiting ED within 30 Days

Within 30 days from discharge, 110 patients (17.4%) re-visited the ED. We found that male gender (*p* value < 0.01), tachycardia (*p* value < 0.01), higher intensity of pain (>3 in numerical pain scale of 0-10) (*p* value < 0.01), hypoalbuminemia (serum albumin < 3.5 gr/dL) (*p* value = 0.03), hyponatremia (*p* value < 0.01), and anemia (as identified as hemoglobin < 13.5 for males, and 12.0 for females) were associated with ED re-visit in 30 days (Table 3). On multivariate analysis, anemia (OR=2.18, 95% CI: 1.12-4.42, *p* value = 0.02), tachycardia (OR = 2.76, 95% CI: 1.27-5.96, *p* value = 0.01) and elevated alkaline phosphatase (OR = 2.61, 95% CI: 1.23-5.57, *p* value = 0.01) were associated with ED revisit within 30 days (Table 4).

## 4. Discussion

Visits to the ED are a major issue in coping with IBD, for both patients and health systems. In the last two decades, the rates of IBD related admissions and IBD related surgeries declined significantly. Surprisingly, the number of IBD-related ED visits has numerically surged, but the proportion of IBD patients who needed ED care decreased [10,13]. This is an interesting phenomenon explained in part by the rise in IBD cases in some parts of the world and by the existence of a group of patients who tend to have recurrent visits to the ED.

Other studies have addressed the ED department visits among IBD patients. Although these studies did not specifically examine the rate of re-visits, they have pointed out several factors to be associated with the risk of any ED visit. Among these risk factors, younger age and shorter duration of disease were risk factors for arrival to the ED [13,14] A study by Nugent and colleagues [11] found that patients who seek care from multiple physicians and patients who use opioids are more likely to attend the ED. Furthermore, studies have shown that while young female patients tend to visit the ED more often [15], older and male patients are more likely to be admitted [12,13]. Smokers, highest income quartile patients, penetrative disease, and steroid exposure are also predictors of hospitalization [10,13].

Several studies have shown that the rates of hospitalization after ED visits decreased along with the reduction in complications and IBD related surgeries [12,13]. Another study from Canada found that despite the decline in rates of hospitalization, rates of ED visits among children with IBD are rising [16]. While this observation of lower rates of hospitalizations can be explained by better control of the disease with newer therapeutic options, the inclining rates of ED visits are yet to be fully understood and pose another challenge suggesting that many needs of IBD patients are still unmet. It suggests that while these patients did not have a major emergency that warrants hospitalization, treatment change, or surgery, these patients have an IBD-related deterioration that should be assessed and managed.

Recurrent visits to the ED have not been thoroughly defined and are probably multifactorial in origin, stemming from undiagnosed flares or complications in the first visit, uncontrolled disease (due to severe disease or unavailability of treatment or ambulatory consultation), uncontrolled pain or irritable bowel syndrome manifestations, among other causes.

In this study, we evaluated the risk factors for recurrent visits to the ED. We found that tachycardia and elevated alkaline phosphatase are associated with re-visiting the ED in both 7 days and 30 days. Hyponatremia and anemia were associated with re-visiting the ED in 7 and 30 days, respectively.

Tachycardia is a nonspecific sign of distress resulting from various pathological and psychological reasons, including infection, inflammation and pain. Along with other inflammatory markers, tachycardia is predictive of urgent findings in computed tomography conducted in the ED [17], but these findings were not consistent in patients with CD attending the ED [18]. Regardless, tachycardia is likely a general sign of distress, and its presence in a patient contemplated to be a candidate for discharge, should warrant re-consideration and possibly implementing further diagnostic or therapeutic measures.

In contrast to a previous study which has shown that patients with IBD who were treated with oral corticosteroids with or without immunosuppressants were more likely to require emergency department visits, in our study, no treatment was associated with recurrent visits to the ED [19].

Anemia is a common comorbidity in IBD, which results from several factors, including blood loss, malabsorption, and systemic inflammation. Anemia is associated with recurrent visits to the ED in 30 days, and it might be a sign of a complication or flare of CD or chronically uncontrolled disease. Besides the fact that patients with uncontrolled disease tend to use emergency department services more often, some fail to have efficient out-patient IBD care, which drives them to re-seek medical care in the ED.

The risk factors identified herein for re-visiting the ED in 7 and 30 days were different than the reported risk factors for visiting the ED and for hospitalization. This points out that recurrent visits to the ED are a distinct problem with a population that differs from the patients who need ED visits or hospitalization.

A study on the utilization of the ED by pediatric IBD patients found that about half of the ED visits can be avoided by optimizing the health care system [20]. Another study emphasized the role of IBD care accessibility in reducing ED visits has shown that, in areas with high accessibility to gastroenterologists, IBD patients were more likely to receive care by specialists and less likely to need ED visits compared to regions with low accessibility to gastroenterologists [21]. A study by Nene et al. [22] investigated acute care IBD patients delivered in either of two distinct pathways: the ED or a rapid access clinic service and found that evaluations conducted by gastroenterologists in the rapid access clinics were rapid and objective and included high rates of C-reactive protein and fecal calprotectin testing, fast-track access to endoscopies, and when needed CT scans, eventually leading to less corticosteroid usage and more optimization of biologic and immunomodulatory therapies. This approach was associated with low rates of ED visits and may be particularly suited for delivering the needed care to ED recurrent patients. This study raises the clinical question of whether intervention in risk factors like anemia and hyponatremia would reduce the chances of re-visiting the ED, or that these are merely markers of uncontrolled disease or an unaddressed aspect of the disease. This question remains unsolved but warrants further investigation.

This study has some limitations. Its retrospective nature, aside from being based on the international classification of diseases-9 (ICD-9) diagnoses which might not be highly accurate. Furthermore, we could not retrieve data regarding the severity of the disease and treatment administered after discharge. As previously mentioned, we excluded frequent visits to the ED in a time range of 90 days from the index visit. As this approach is intended to exclude frequent visits for the same unresolved reason from affecting the results, it has the potential disadvantage of excluding unrelated visits that preferentially would have been included in the analysis.

In conclusion, this study highlights the risk factors of recurrent visits to the ED among patients with CD. If a patient is intended to be discharged from the ED, we advise special attention to be paid to those who present with tachycardia, elevated alkaline phosphatase, hyponatremia and anemia as these patients are most expected to re-visit the ED. A second consideration of the discharge decision, a search for unsolved problems and a trial of addressing the unmet needs of these patients may prevent recurrent visits to the ED. This study offers an opportunity to characterize this special population of patients, which may be a focus of future studies to evaluate strategies to reduce recurrent ED visits.

## Figures and Tables

**Table 1:** Characteristics of the study population and controls. Re-visits after 7 days.

Variable	Study Population *n* = 632	No Recurrent ED Visit in 7 Days (*n* = 579)	Recurrent ED Visit within 7 Days (*n* = 53)	*p*-Value
Gender (Male)	279 (47%)	45.8%	60.4%	0.045
Age (y)	36 (26, 48.7)	36 (25, 49)	39 (31.5, 47.5)	0.19
**Comorbidities**				
Smoker	14 (2.2%)	2.4%	0.0%	0.252
Hyperlipidemia	31 (4.9%)	4.8%	5.7%	0.790
Hypertension	31 (4.9%)	6.9%	7.5%	0.861
Diabetes mellitus	33 (5.2%)	5.4%	3.8%	0.621
**IBD treatments**				
Prednisone	60 (9.5%)	9.2%	13.2%	0.335
Biologics	122 (19.3%)	19.7%	15.1%	0.473
immunomodulators	100 (15.8%)	16.1%	13.2%	0.697
**Time of ED visits**				
day of arrival (Sunday–Thursday)	503 (79%)	80.1%	73.6%	0.285
morning shift	323 (51.1%)	51.8%	43.4%	0.413
afternoon shift	212 (33.5%)	32.8%	41.5%	
night shift	97 (15.3%)	15.4%	15.1%	
**Clinical features**				
Tachycardia	15.2%	13.8%	30.2%	0.001
Fever	36.8 ± 0.4	36.8 ± 0.38	36.8 ± 0.43	0.65
Pain > 3	45.1%	44.0%	57.1%	0.134
**Laboratory tests**				
WBC	9.3 ± 3.1	9.20 ± 3.11	10.15 ± 3.27	0.501
Anemia	42.4%	42.0%	47.2%	0.463
PLT	266 (219, 335)	267 (221, 338)	257 (212, 318)	0.531
Creatinine	0.8 (0.67, 0.96)	0.8 (0.67, 0.96)	0.82 (0.69, 0.93)	0.49
Hypoalbuminemia	15.6%	14.6%	25.0%	0.124
Bilirubin	0.48 (0.34, 0.67)	0.48 (0.34, 0.66)	0.49 (0.32, 0.79)	0.49
Elevated ALP	17.1%	15.8%	30.6%	0.009
Abnormal AST	16.9%	16.6%	20.8%	0.44
Abnormal ALT	17.2%	16.9%	20.8%	0.48
LDH	186 (155.5, 228)	184.0 (155.0, 225.0)	212.0 (164.0, 254.0)	0.086
Hyponatremia	13.4%	12.0%	28.3%	0.001
CRP	11.0 (3.9, 26.0)	10.9 (3.8, 26.9)	12.1 (7.6, 19.6)	0.581

Tachycardia, Pulse > 100 bpm; fever, temperature > 38.1 degrees Celsius; anemia, hemoglobin < 13.5 gr/dL for males, and 12.0 gr/dL for females; hypoalbuminemia, serum albumin < 3.5 gr/dL; elevated ALP, alkaline phosphatase > 120 U/L; abnormal ALT, alanine transaminase > 40 U/L; abnormal AST, aspartate transaminase > 40 U/L; hyponatremia, sodium < 135 mEq/L. ED, emergency department; IBD, inflammatory bowel diseases; WBC, white blood cells; PLT, Platelets; LDH, lactate dehydrogenase; CRP, C-reactive protein.

**Table 2:** Multivariate analysis of parameters associated with re-visiting the ED in 7 days.

	OR	95% CI		*p*-Value
Tachycardia	2.194	1.111	4.334	0.024
Elevated ALP	2.088	1.070	4.072	0.031
Hyponatremia	2.516	1.241	5.102	0.010

Tachycardia, Pulse > 100 bpm; elevated ALP, alkaline phosphatase > 120 U/L; hyponatremia, sodium < 135 mEq/L. OR, odds ratio; CI, confidence interval.

**Table 3:** Characteristics of the study population and controls.

Variable	Study Population *n* = 632	No Recurrent ED Visit in 30 Days (*n* = 531)	No Recurrent ED Visit in 30 Days (*n* = 101)	*p*-Value
Gender (Male)	279 (47%)	43.9%	63.4%	<0.001
Age (year)	36 (26, 48.7)	36 (25, 47)	39 (28, 50)	0.09
**Comorbidities**				
Smoker	14 (2.2%)	2.4%	1.0%	0.710
Hyperlipidemia	31 (4.9%)	1.1%	2.0%	0.620
Hypertension	31 (4.9%)	6.6%	8.9%	0.401
Diabetes mellitus	33 (5.2%)	5.1%	5.9%	0.723
IBD treatments				
Prednisone	60 (9.5%)	8.7%	13.9%	0.102
Biologics		20.0%	15.8%	0.336
Immunomodulators		16.4%	12.9%	0.375
**Time of ED visits**				
Day of arrival (Sunday–Thursday)	503 (79%)	80.8%	73.3%	0.086
Morning shift	323 (51.1%)	52.7%	42.6%	0.064
Afternoon shift	212 (33.5%)	31.6%	43.6%	
Night shift	97 (15.3%)	15.6%	13.9%	
**Clinical features**				
Pulse > 100 (bpm)	15.2%	12.9%	27.0%	<0.001
Fever	36.8 ± 0.4	36.8 ± 0.38	36.87 ± 0.41	0.180
Pain > 3	45.1%	42.4%	60.0%	0.009
**Laboratory tests**				
WBC	9.3 ± 3.1	9.24 ± 3.15	9.47 ± 3.06	0.501
Anemia	42.4%	40.5%	52.5%	0.025
PLT	266 (219, 335)	265 (221, 332)	269 (215, 362)	0.511
Creatinine	0.8 (0.67, 0.96)	0.79 (0.67, 0.95)	0.69 (0.83, 0.96)	0.123
Hypoalbuminemia	15.6%	13.6%	25.5%	0.027
Bilirubin	0.48 (0.34, 0.67)	0.48 (0.34, 0.66)	0.46 (0.33, 0.73)	0.919
Alkaline phosphatase > 120	17.1%	14.9%	28.3%	0.002
Abnormal AST	16.9%	16.4%	19.8%	0.40
Abnormal ALT	17.2%	16.9%	18.8%	0.65
LDH	186 (155.5, 228)	185.0 (155.7, 225.0)	189.0 (149.0, 250.0)	0.624
Hyponatremia	13.4%	11.8%	21.8%	0.007
CRP	11.0 (3.9, 26.0)	10.6 (3.8, 25.8)	12.4 (7.7, 28.9)	0.341

Tachycardia, Pulse > 100 bpm; fever, temperature > 38.1 degrees celsius; anemia, hemoglobin < 13.5 gr/dL for males, and 12.0 gr/dL for females; hypoalbuminemia, serum albumin < 3.5 gr/dL; elevated ALP, alkaline phosphatase > 120 U/L; abnormal ALT, alanine transaminase > 40 U/L; abnormal AST, aspartate transaminase > 40 U/L; hyponatremia, sodium < 135 mEq/L. ED, emergency department; IBD, inflammatory bowel diseases; WBC, white blood cells; PLT, Platelets; LDH, lactate dehydrogenase; CRP, C-reactive protein.

**Table 4:** Multivariate analysis of parameters associated with re-visiting ED in 30 days.

	OR	95% CI		*p*-Value
Tachycardia	2.756	1.275	5.958	0.010
Anemia	2.177	1.118	4.240	0.022
Elevated alkaline phosphatase	2.614	1.226	5.573	0.013
Pain intensity >3 (in numeric pain intensity scale 1–10)	1.892	0.894	4.006	0.095

Tachycardia, Pulse > 100 bpm; anemia, hemoglobin < 13.5 gr/dL for males, and 12.0 gr/dL for females; elevated ALP, alkaline phosphatase > 120 U/L. OR, odds ratio; CI, confidence interval.
